# Psychological distance affects real movements in virtual reality: distance to food in anorexia nervosa

**DOI:** 10.1186/s40337-025-01357-0

**Published:** 2025-08-06

**Authors:** Mechteld M. van den Hoek Ostende, Giulia Brizzi, Valentina Meregalli, Philipp A. Schroeder, Enrico Collantoni

**Affiliations:** 1 Department of Psychology, Clinical Psychology & Psychotherapy, University of Tübingen, Tübingen, Germany; 2 Department of Psychology, Università Cattolica del Sacro Cuore, Milan, Italy; 3 Humane Technology Laboratory, Università Cattolica del Sacro Cuore, Milan, Italy; 4 Department of Neuroscience, University of Padova, Padova, Italy; 5 Department of General Psychology, University of Padova, Padova, Italy; 6 German Center for Mental Health (DZPG) partner site Tübingen, Tübingen, Germany

**Keywords:** Anorexia nervosa, Construal-level theory, Virtual reality, Low-calorie food, Modality

## Abstract

**Background:**

Patients with restrictive Anorexia Nervosa (AN-R) severely restrict their food intake, often showing significant food avoidance behavior, especially for diet-goal threatening and high-calorie foods. Still, stringent comparisons of avoidance behaviors in relation to calorie dense foods, low-calorie food and abstract (amodal) food cues are required to better understand the underlying mechanisms.

**Methods:**

Approach-avoidance behavior was measured in individuals with AN-R (*n* = 21) and Healthy Controls (HC; *n* = 19) using a virtual reality stop-signal task. In a virtual environment, participants had to reach a digitally rendered hand toward low-calorie, high-calorie and amodal (packaged) food cues, as well as nonfood cues (shoes). If a stop-sign appeared, they had to inhibit this movement (stop-trials). They also rated how much they liked and wanted each stimulus on a visual analog scale from 0 to 100.

**Results:**

Participants showed more approach behavior towards amodal food cues compared to high-calorie concrete food cues (*t*[39] = 25.38, *p* <.001, *d* = 4.01). Furthermore, patients with AN-R reported lower wanting for high-calorie foods (*t*[37] = 2.13, *p* =.040, *d* = 2.13) and greater wanting for nonfood cues (*t*[37] = -3.35, *p* =.002, *d* = 3.35). Across groups, liking was highest for high-calorie food, both packaged (*t*[39] = 4.03, *p* =.002, *d* = 0.40) and unpackaged (*t*[39] = 3.53, *p* =.007, *d* = 0.36).

**Conclusions:**

Food presentation can influence approach behavior toward food cues. Future research is needed to determine whether the use of abstract food cues can facilitate food approach behavior in individuals with AN-R.

**Supplementary Information:**

The online version contains supplementary material available at 10.1186/s40337-025-01357-0.

## Abstract (plain language summary)

Individuals with anorexia nervosa (AN) tend to avoid calorie-dense foods. This avoidance also extends to food images. However, we do not fully understand which features of food lead to avoidance. In this article, we examine how four different categories - high-calorie food in packaging, high-calorie food unpackaged, low-calorie food unpackaged and nonfood (shoes) - affect avoidance in patients with AN compared to healthy control participants. We found that unpackaged foods were avoided more than packaged foods, but that packaged and unpackaged high-calorie foods were equally wanted and liked by patients with AN. This could indicate that packaged foods are easier to access for individuals with AN. If so, making food more abstract may make it easier for individuals with AN to interact with it. Future studies should thus test if using more abstract food cues could help individuals with AN reduce their avoidance of food cues all together.

**Supplementary Information:**

The online version contains supplementary material available at 10.1186/s40337-025-01357-0.

## Introduction

Anorexia nervosa (AN) is an eating disorder that is characterized by severe restriction of food intake, fear of gaining weight and altered body perception [1]. A hallmark of AN, particularly the restrictive subtype (AN-R), is the deliberate avoidance of calorie-dense, high-fat foods, leading to a highly selective and restrictive eating pattern [2]. This maladaptive food choice is consistently observed in patients and contributes to poor long-term outcomes, including high relapse rates and significant morbidity [2, 3].

Understanding the mechanisms underlying these restrictive food choices is crucial for developing effective interventions. One perspective suggests that individuals with AN-R exhibit aberrant food valuation processes, where high-calorie foods are undervalued owing to altered neural mechanisms involved in decision-making [3]. Other perspectives suggest that the severe dietary restriction observed in AN may be closely linked to cognitive adaptations that foster approach-avoidance conflicts toward food cues (e.g., [4, 5–8]) and reduced impulsivity (e.g., [9–11]). In particular, research has shown that individuals with AN, especially those with the restrictive subtype (AN-R), rely heavily on cognitive control mechanisms to maintain their rigid eating behaviors [12]. Over time, these control mechanisms appear to shift toward more automatic, habit-like processes. This further reinforce behaviors such as food avoidance and the suppression of impulsive drives, despite the significant physical and psychological demands these behaviors impose [7, 13]. This transition from conscious cognitive control to habitual patterns suggests that the restrictive behaviors in AN become increasingly difficult to alter, as they are no longer driven solely by active decision-making but rather by ingrained, automated processes. While research has extensively documented these cognitive shifts, little is known about the underlying mental representations that support this progression toward behavioral inflexibility.

Construal level theory [14] proposes that environmental cues or events can be interpreted at different construal levels. If a situation is interpreted at a higher construal level, the psychological distance to the situation becomes greater, increasing the activation of related abstract concepts. When interpretation occurs at a lower construal level, the psychological distance is decreased, which in turn activates concrete and sensory aspects of the stimuli. For example, in line with construal level theory when pizza is represented at a higher construal level, abstract information such as “edible” is activated. When it is represented at a lower construal level, on the other hand, sensory information such as “savory” is activated. According to modal and amodal theory of mental representation [15], interpretation at a higher construal level may lead to less tempting, amodal representations of the food environment, whereas lower levels would lead to tempting, modal representations.

Moreover, dual-process models (e.g., [16]) state that higher construal levels are more closely related to effortful mental processes such as inhibitory control. Lower construal levels, on the other hand, are associated with fast, automatic processes such as impulsive reactions. Returning to our example, the sensory properties of pizza activated at lower construal levels is thought to be more tempting and elicit unreflected approach behavior. Combined, these theories therefore suggest that lower construal levels could be associated with more impulsive behavior toward tempting food cues, and therefore also with reduced inhibitory control over food intake. As higher construal levels should be closer to the information used to process inhibitory control, it also suggests that food information represented at a higher construal level could be associated with increased control over food intake. Indeed, priming and cuing of higher-level construal thinking are associated with lower (unhealthy) snack preference and intake [17–19]. Furthermore, patients with AN demonstrate lower delay discounting [9–11], which means that they have an increased ability to delay rewards. This is often interpreted to reflect increased self-control over food intake in AN [9–11]. With regard to higher construal level thinking, previous work links the ability to abstractly represent the delayed object to lower delay discounting [20, 21]. It is therefore conceivable that higher construal level processing might also assist patients with AN-R in their avoidance of (high-calorie) food. Accordingly, avoidance of food cues [6, 7, 22] may reflect the activation of higher construal levels pertaining to dieting and weight loss goals by threatening (high-calorie) food stimuli.

One way to effectively examine the effect of construal level on approach-avoidance behavior may be through virtual reality (VR). One study demonstrated that food stimuli in a virtual environment elicit a similar emotional response in patients with eating disorders as real food [23]. Moreover, the same study showed stronger emotional responses for both virtual reality food and real food compared to food pictures. Further studies have since backed the advantage of virtual reality food over food pictures, indicating that VR allows for a more ecologically valid method of measuring reactions to food stimuli than paradigms using food pictures [23–26]. Moreover, virtual environments are flexible and allow for the implementation of more abstract food stimuli, such as food packaging with labeled content. Additionally, VR allows for fine-grained monitoring of hand movement trajectories. This has, for instance, been used to demonstrate avoidance in social anxiety [27] and spider phobia [28]. In the domain of disordered eating, motion tracking in 2D space has already demonstrated reduced motor distractibility caused by high-calorie food cues [29]. Furthermore, Schroeder, Collantoni, et al. [7] reported evidence for avoidance of food items by AN-R patients in a VR kinematic (stop) task. During this task, participants reached for food and nonfood items placed at approximately arm-lengths. Because participants had to inhibit their approach response in a subset of trials in which a stop signal was presented, this setup allows monitoring of food approach and inhibition [7, 30]. In this task, unlike healthy controls, patients with AN-R did not reach as far toward food as nonfood items in both go- and stop-trials, thus displaying avoidance of food cues. However, the study did not differentiate between the calorie content of the food stimuli, which may moderate behavioral avoidance in AN-R [29]. Moreover, the concreteness of the food stimuli, especially in VR, may have acted as a cue for higher-construal thinking in AN-R, enabling reduced approach behavior. The inclusion of more abstract cues may therefore reduce avoidance behavior.

In summary, AN-R is characterized by severe restriction of food intake, which is reflected in increased food avoidance. It remains unclear, however, if this is specific to more threatening, high-calorie foods and whether this effect is related to the representation of the stimulus. Therefore, the current study employed amodal (packaged/named) high-calorie stimuli in addition to the same high-fidelity, high-calorie, low-calorie, and nonfood (shoe) stimuli in the kinematic VR paradigm introduced by Schroeder, Collantoni, et al. [7]. We expected response trajectories to be longer for go trials than for stop trials for all participants. Moreover, we expected that AN-R patients would demonstrate a general avoidance of food cues. This should be evident in shorter trajectories for AN-R patients than for controls in trials with high-calorie and low-calorie food stimuli. Finally, we explored whether stimulus presentation at a higher construal level (amodal) could reduce AN-R patients’ advantage in food avoidance.

## Methods

### Participants

Patients with acute AN-R were recruited from the Eating Disorder Unit of the Hospital of Padova and the affiliated Eating Disorder Unit of the San Bortolo Hospital of Vicenza. Healthy controls were matched for age and recruited through direct contact with the experimenters in North Italy. We ran a power analysis for a medium effect size (*d* = 0.5) for the contrasts of interest. To obtain a power of 1-β = 0.9 with a significance level of α = 0.05, we required *N* = 30 participants per group. Full data were obtained from 54 participants, 14 of whom dropped out because they did not have sufficient successful stop trials (i.e., fewer than 10% correct stops). In the patient group, participants that dropped out did not differ significantly in key demographic features from included participants (Table S1). In the control group, drop-outs were significantly older and demonstrated lower trait anxiety than included participants (Table S2). The final sample consisted of 21 patients with acute AN-R according to the DSM-5 [1] and 19 healthy controls (HCs). By acute AN-R we refer to patients diagnosed with AN, restrictive subtype, currently in a state of malnutrition and actively receiving multidisciplinary treatment. All patients had a BMI below 18 kg/m², and none were in partial or full remission according to DSM-5 criteria. The exclusion criteria for both groups were age under 14 years, self-reported diagnosis of neurological disorders, and self-reported diagnosis of psychosis. Additionally, healthy controls were excluded if they had a current or lifetime diagnosis of an eating disorder in accordance with DSM-5 criteria. We exclusively recruited female patients and HC due to low availability of male AN-R patients. Since AN in males is associated with different etiological and maintenance factors [31, 32], inclusion of a small number of participants would have complicated the interpretation of the data. All participants provided informed consent. For underage participants, informed consent was also obtained from their legal guardians. The local ethics committees approved the current study (protocol number: 1831).

The study was conducted in accordance with the Declaration of Helsinki and was preregistered on the Open Science Framework (https://osf.io/wuphk). Data and scripts are available at https://osf.io/acxkg.

### Experimental procedure

A brief and ambulatory VR experiment was conducted in the respective clinics. The AN-R group was tested in their respective hospital clinic, whereas healthy controls participated at the laboratory facilities of the University of Padova. The study environments were quiet, with ample space and lighting to allow for movement tracking. The participants were seated in a chair for the duration of the experiment. To provide a similar environment to all the participants, the VR environment was adapted to the participants’ individual seating height. The participants received instructions on the task and completed 10 practice trials. The participants then completed the VR task (30 min). At the end of the task, the participants rated all stimuli on wanting and liking scales and filled out the questionnaires.

### Questionnaires

All the participants completed a demographic questionnaire on their age, nationality, years of education, time since their last meal in hours, current hunger level (7-point Likert scale) and current medication. The participants’ body mass index (BMI) was either extracted from their medical records (AN-R) or calculated from self-reported weight and height (HC). To assess participants’ eating disorder psychopathology, participants completed the Eating Disorder Examination Questionnaire (EDE-Q; [33]). This self-report questionnaire consists of 28 items that are answered on a 7-point Likert scale. The EDE-Q has good-to-excellent psychometric properties. Eating disorder psychopathology is represented by a global score (current data: Cronbach’s α = 0.98), as well as four subscales: restraint (α = 0.91), eating concern (α = 0.88), weight concern (α = 0.94), and shape concern (α = 0.97). Additionally, we wanted to further characterize our sample with regards to dimensions that can be related to approach (i.e., impulsivity) and avoidance behavior (i.e., anxiety; [34]), To this end, we measured impulsivity with the UPPS Impulsive Behavior Scale (subscales: negative urgency, lack of perseverence, lack of premeditation, sensation seeking and positive urgency; [35, 36]) and anxiety with the State-Trait Anxiety Inventory (STAI; [37]), which had Cronbach’s α = 0.71 and α = 0.65, respectively. All included questionnaires have been validated in Italian.

### Stimuli

We used 20 stimuli of different 3D objects: 5 high-calorie foods, 5 low-calorie foods, 5 amodal (packaged, high-calorie) foods, and 5 nonfood images. For the nonfood category, we used objects that represented shoes; this category has as an advantage that it is positively valenced and that, opposed to other neutral stimuli (e.g., office supplies) the items are generally desirable to the current sample [7]. In previous research on construal level theory, words were generally used to represent psychologically distant items [38, 39]. To provide a more similar stimulus complexity, neutral, brown-bag packaging with a food name label was employed for all amodal foods (i.e., *panini*,* pizza*,* burger*,* brioche*,* torta*). The names for amodal foods corresponded to the category of high-calorie foods. All modal stimuli were photorealistic scans drawn from open asset libraries (cf. [7, 30]). The high-calorie foods and nonfood images were taken from a previous study [7]. The links to the low-calorie foods and the packaging for the amodal stimuli are reported in the supplements. The stimuli were adjusted to be size-consistent within and between categories and piloted internally in a small sample of healthy volunteers.

### Stimulus ratings

All stimuli were shown individually at the end of the experiment and evaluated within VR on liking and wanting. Whereas we asked participants for subjective liking in the same manner, we operationalized wanting as the degree to which the participants wanted to interact with the object. For the food stimuli we further specified this interaction to be eating (opposed to, e.g. cooking). The participants used the VR hand controller to rate all stimuli on liking and wanting on a visual analog scale (from 0 [not at all] to 100 [a lot]) at the end of the task. During the rating, stimuli were shown with continuous rotation to view all the angles.

**Fig. 1:**
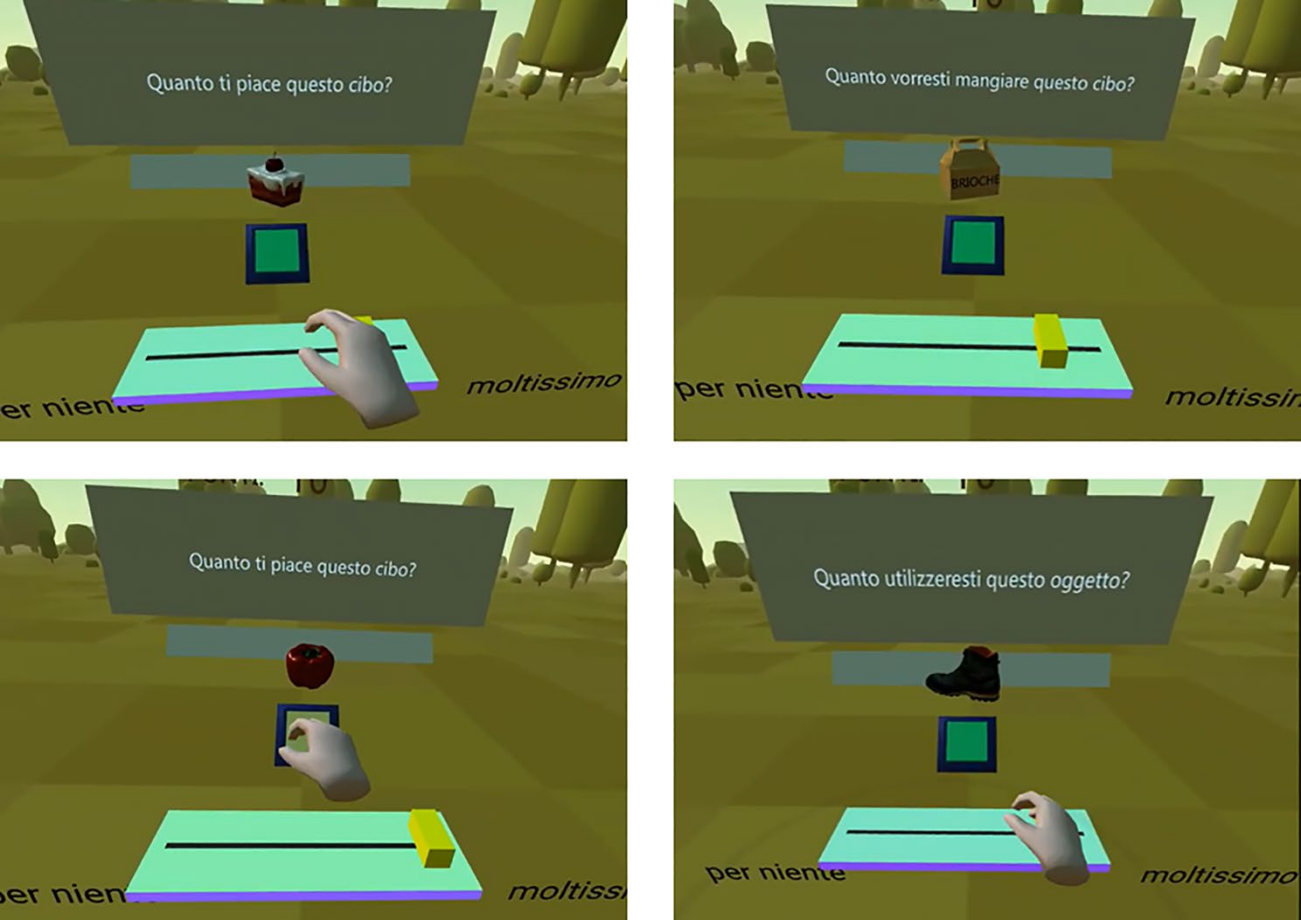
Experimental environment. Environment in which the participants rated stimuli on wanting (“Quanto utilizzeresti questo oggetto?” [How much do you want to use this object]/”Quanto vorresti mangiare questo cibo?” [How much do you want to eat this food? ]) and liking (“Quanto ti piace questo oggetto?” [How much do you like this object]/”Quanto ti piace questo cibo?” [How much do you like this food? ]) on visual analog scales. Each frame contains an example of a stimulus type (high-calorie, amodal, low-calorie, or nonfood).

### Virtual reality task

We adapted the VR task from Schroeder, Collantoni, et al. [7]. The VR task required participants to tap objects presented in front of them in the virtual environment. The VR task was run on the PICO 4 All-in-One VR Headset (Beijing Bytedance Technology LLC, Peking, China). The PICO 4 controller was displayed as a white right hand in the virtual environment, the position and movement of which were updated in accordance with the sampling rate. We instructed the participants to reach for the objects in a direct motion without interrupting their movement in the go trials. The participants started the trials by moving their hand to the starting position, indicated by a gray cube, which turned green at trial initiation. In the virtual environment, stimuli were placed approximately 65 cm from the starting position, with random variation in the vertical (10 cm) and horizontal (5 cm) directions. Since response detection depended on the hand and stimulus colliders, the effective movement distance to reach the stimulus was approximately 30 cm.

In 25% of the trials, a stop signal in the form of a virtual “stop” traffic sign appeared behind the stimulus (stop trials). The stop signal appeared 150 cm away from the participant. In these trials, the participants were instructed to refrain from touching the object. The timing of the stop signal and the Stop Signal Delay (SSD) were determined dynamically. The SSD started once the participants initiated movement away from the starting position. The SSD was subsequently adapted according to the participants’ success at inhibiting themselves: if participants were successful at inhibiting themselves, the SSD decreased by 40 ms to make the task more challenging, whereas it increased by 40 ms after failed inhibition. The initial SSD was set at 200 ms.

The VR task consisted of 400 trials (100 stop trials) in randomized order. Ahead of the task, the participants completed 5 practice trials without the stop signal, followed by 5 practice trials with the stop signal. The participants were given the opportunity to ask questions after the practice trials. During the task, participants were given error feedback if they were reacting too slowly (Italian “*Per favore rispondi più veloce*”/“please respond faster” for responses exceeding 1500 ms) or if they made mistakes (Italian “*Non è corretto*”/“that was not correct”).

### Data processing

We continuously recorded the hand position at a mean sampling rate of 62 Hz. To accommodate variable sampling rates, we used interpolation and time standardization, as in previous studies [7, 30]. We used the *mousetrap* package in R to extract motion parameters. Since this allowed us to analyze the movement in 2D space only, we additionally used custom MATLAB scripts to validate our results in 3D space [40]. We obtained the same results for both extraction measures and therefore only report on the analyses of the variables extracted by mousetrap. The trajectories were centered to start at the same starting point (0,0,0) to control for small trial-by-trial and participant-specific deviations. The maximum depth position was determined as the most extreme hand position away from the starting point.

### Data analysis

Data processing and statistical analysis were performed in R [41] via the *tidyverse* package [42]. We compared demographic variables through independent samples *t*-tests. A significant difference emerged between the groups in the time since the last meal was consumed. Because fasting state can influence inhibitory control [43, 44], the *time since the last meal* was used as a covariate in our analyses. To determine the effects on participants’ approach behavior (i.e., the maximum depth position), we therefore used a 2 (Group: AN-R, HC) × 2 (Trial type: go trial, stop trial) × 4 (Stimulus type: high-calorie food, low-calorie food, amodal food, shoe) analysis of covariance (ANCOVA). Similarly, we used the same 2 × 2 × 4 ANCOVA for movement initiation times. As false alarms only apply to stop trials, whereas reaction time is meaningful only for go trials, these secondary outcome measures were analyzed with a 2 (Group: AN-R, HC) × 4 (Stimulus type: high-calorie food, low-calorie food, amodal food, shoe) ANCOVA. Furthermore, we used a 2 (Group: AN-R, HC) × 4 (Stimulus type: high-calorie food, low-calorie food, amodal food, shoe) ANCOVA to analyze participants’ liking and wanting ratings of the presented stimuli. As normality was violated for the stimulus ratings, we additionally used linear mixed models to validate our results. Model comparisons did not indicate different effects from the here presented ANCOVA’s. Significant main and interaction effects that included stimulus type were followed-up on the basis of preregistered contrasts: high-calorie and nonfood, high-calorie and low calorie, and amodal and nonfood. Greenhouse–Geisser correction was applied when the assumption of sphericity was violated. Exploratorily, we also compared amodal and high-calorie stimuli.

## Results

### Group characteristics

AN-R patients and HCs did not differ in terms of age or UPPS score. As expected, AN-R patients had a lower BMI and higher EDE-Q scores than HCs did, as well as greater trait anxiety. However, the AN-R group was also less hungry and ate more recently than the HCs did, which was therefore taken up as a covariate in our subsequent analyses (Table 1).

**Table 1:** Demographic and clinical characteristics of the sample

Variable	Group		Group comparison
	AN-R (*n* = 21)	HC (*n* = 19)	
Age	17.1 (2.86)	18.1 (4.37)	*t*(38) = 0.87, *p* =.388
BMI	16.4 (1.22)	21.2 (1.78)	*t*(38) = 9.94, *p* <.001
Hunger	1.35 (0.93)	3.21 (1.90)	*t*(38) = 3.91, *p* <.001
Time since last meal	1.23 (0.72)	2.89 (2.27)	*t*(38) = 3.18, *p* =.003
EDE-Q total	3.88 (1.33)	0.84 (0.89)	*t*(38) = -8.40, *p* <.001
EDE-Q restraint	3.13 (1.72)	0.24 (0.55)	*t*(38) = -7.02, *p* <.001
EDE-Q eating concern	3.39 (1.16)	0.53 (1.08)	*t*(38) = -8.07, *p* <.001
EDE-Q shape concern	4.81 (1.33)	1.37 (1.16)	*t*(38) = -8.71, *p* <.001
EDE-Q weight concern	4.17 (1.64)	1.21 (1.06)	*t*(38) = -6.70, *p* <.001
STAI trait	63.5 (8.47)	49.3 (8.54)	*t*(38) = -5.28, *p* <.001
UPPS total	8.40 (1.77)	9.51 (2.08)	*t*(38) = 1.82, *p* =.077
UPPS negative urgency	10.5 (2.75)	11.4 (2.27)	*t*(38) = -1.18, *p* =.246
UPPS lack of perseverance	7.00 (2.57)	6.68 (2.47)	*t*(38) = 0.395, *p* =.695
UPPS lack of premeditation	6.57 (2.06)	7.79 (2.76)	*t*(38) = -1.59, *p* =.120
UPPS sensation seeking	9.67 (2.96)	11.0 (3.38)	*t*(38) = -1.33, *p* =.191
UPPS positive urgency	8.29 (2.51)	10.6 (2.99)	*t*(38) = -2.70, *p* =.010

AN-R = patients diagnosed with anorexia nervosa, restrictive type; BMI = body mass index; EDE-Q = Eating Disorder Examination Questionnaire; HC = healthy controls; STAI = State-Trait Anxiety Inventory; UPPS = UPPS Impulsive Behavior Scale

### All participants reach furthest to amodal stimuli

**Fig. 2:**
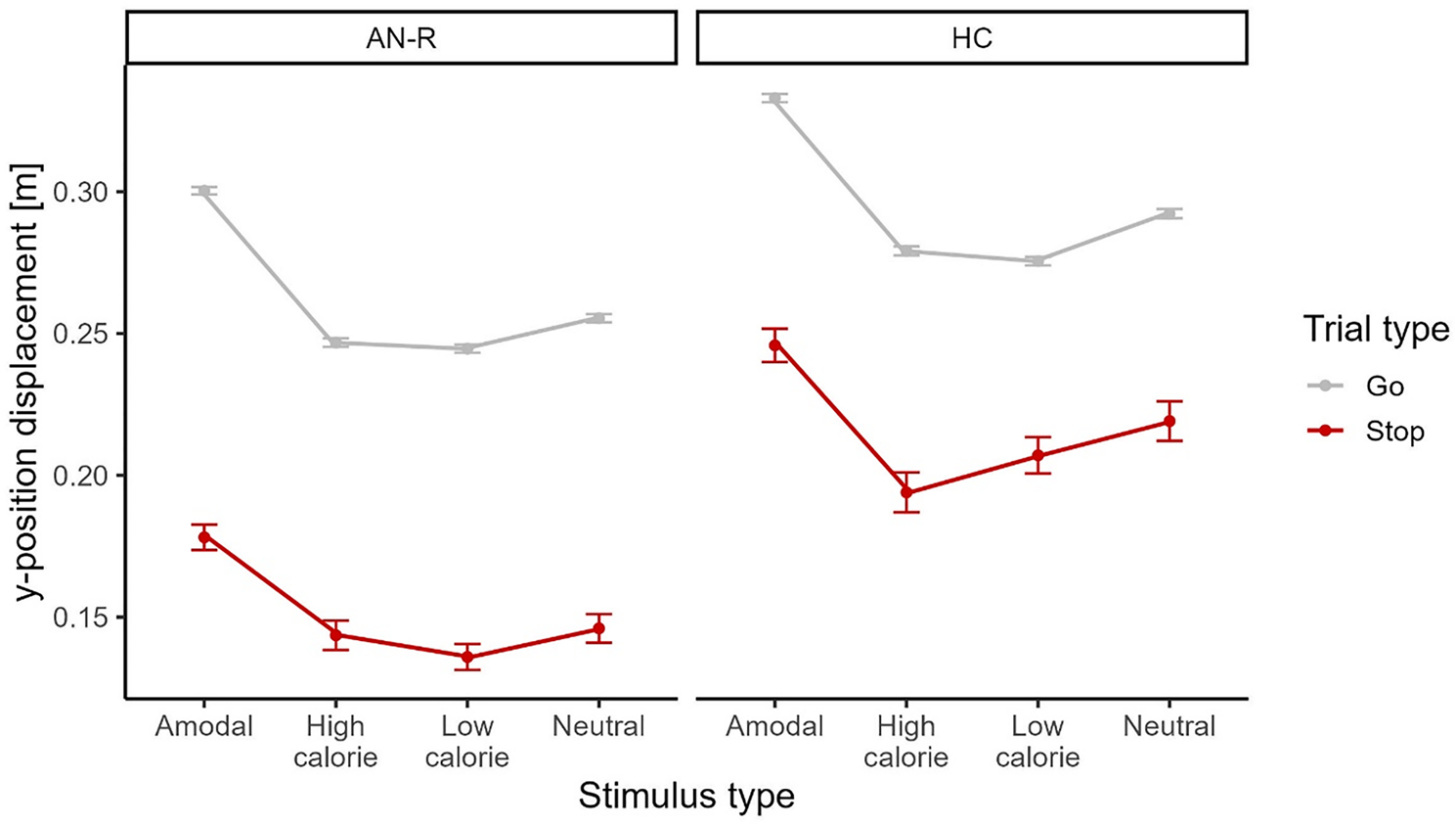
Y-position displacement in successful trials as a function of stimulus type, trial type and group. The participants in the healthy control (HC) group reached further than did the participants in the anorexia nervosa, restrictive type (AN-R) group did. All participants reach furthest for amodal food stimuli. The error bars indicate standard errors

Our analyses revealed all participants reached further towards amodal than towards nonfood and high-calorie food trials. Furthermore, all participants reached further to neutral than high-calorie stimuli. Specifically, to determine the dependence of approach behavior on Group, Trial type and Stimulus type, we performed a mixed ANCOVA on all accurate trials with *time since the last meal* as a covariate. Analyses revealed main effects of Group (*F*[1,37] = 15.63, *p* <.001, η^2^ = 0.19), Trial type (*F*[1,37] = 106.62, *p* <.001, η^2^ = 0.15), and Stimulus type (*F*[1,111] = 107.05, *p* <.001, η^2^ = 0.50) in the absence of interaction effects (all *F*s < 2.14, *p*s > 0.10; Fig. 2). As expected, participants reached further on successful go trials than stop trials. The main effect of Group constituted shorter approach distances in the AN-R group than in the HC group.

We followed up on the main effect of Stimulus type by using paired sample *t*-tests to compare our predefined contrasts of interest. Our analyses revealed differences between high-calorie and nonfood stimuli (*t*[39] = -6.43, *p* <.001, *d* = 1.02), amodal and nonfood stimuli (*t*[39] = 18.08, *p* <.001, *d* = 2.86) and amodal and high-calorie stimuli (*t*[39] = 25.38, *p* <.001, *d* = 4.01). There was no difference in the maximum approach between high- and low-calorie stimuli (*t*[39] = 1.63, *p* =.111).

### High-calorie and amodal foods elicit similar levels of liking

**Fig. 3:**
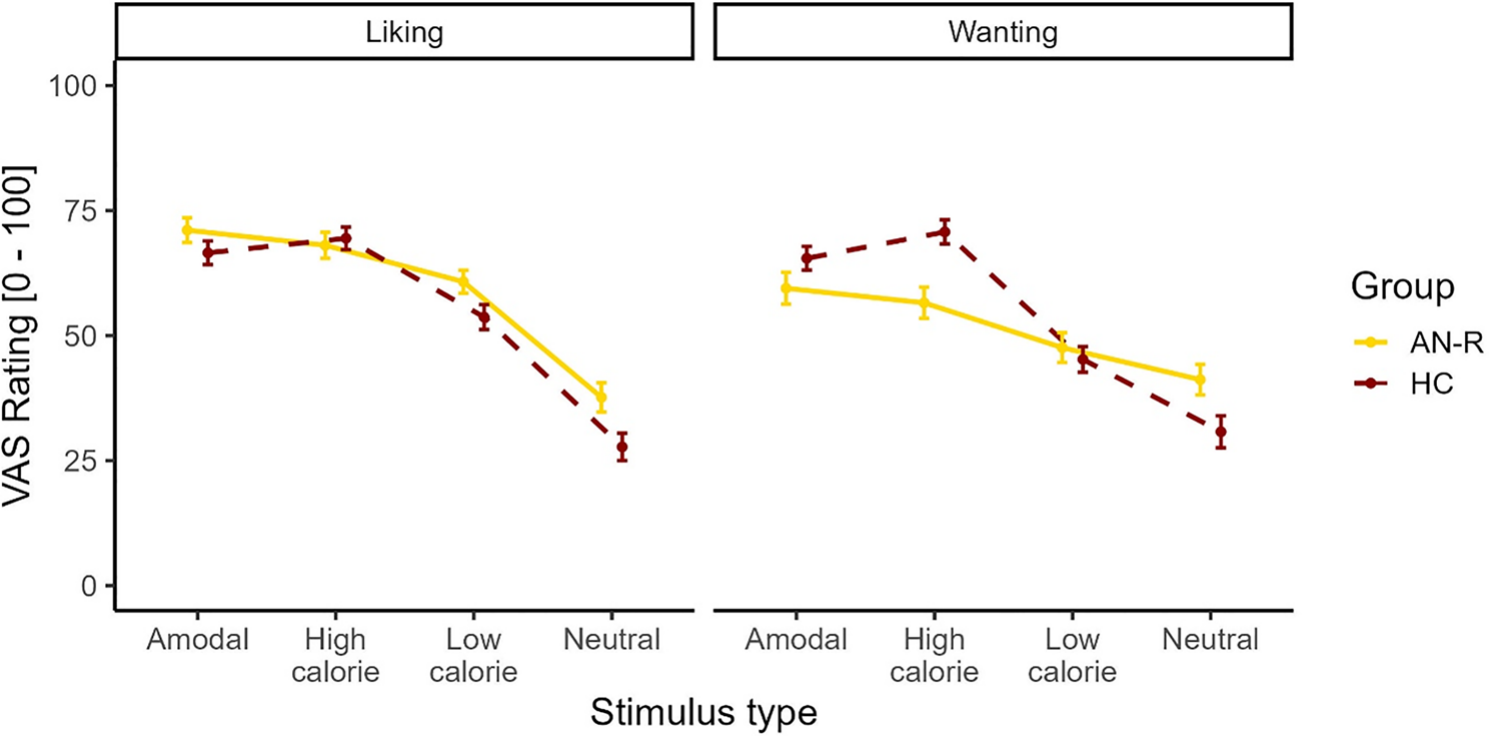
Wanting and liking ratings of each stimulus type rated on Visual Analog Scales (VAS). Note Liking ratings were similar between anorexia nervosa, restrictive type (AN-R), and healthy controls (HC). HC reported wanting high-calorie food more than AN-R did, whereas AN-R reported wanting nonfood cues (shoes) more than HC did. The error bars indicate standard errors

A mixed ANCOVA revealed a significant main effect of Stimulus type (*F*[2.08, 76.92] = 32.38, *p* <.001, η^2^ = 0.314; see Fig. 3). There were no significant group differences in food liking (*F*[1, 37] = 2.90, *p* =.097) or two-way interaction with Stimulus type (*F*[2.08, 76.92] = 1.62, *p* =.203). We subsequently performed post-hoc analysis to compare the responses for the different stimulus types. The liking ratings of nonfood were lower than the ratings of all the food stimuli (*t*[39] > 6.60, *p* <.001). Moreover, low-calorie foods were liked less than high-calorie food (*t*[39] = 3.53, *p* =.007, *d* = 0.36) and amodal food stimuli (*t*[39] = 4.03, *p* =.002, *d* = 0.40). High-calorie and amodal food stimuli elicited similar levels of liking (*t*[39] = 0.12, *p* = 1.00; Fig. 3).

### Patients have blunted wanting for high-calorie sensory, but not amodal, food cues

Regarding wanting ratings, we found that patients with AN-R wanted high-calorie stimuli less than healthy controls. This difference did not transfer to the amodal, boxed high-calorie stimuli. Specifically, mixed ANCOVA with wanting ratings revealed a significant main effect of Stimulus type (*F*[1.64, 60.59] = 22.83, *p* <.001, η^2^ = 0.246) and a significant interaction effect of Group × Stimulus type (*F*[1.64, 60.59] = 6.64, *p* =.004, η^2^ = 0.087). There was no main effect of Group (*F*[1, 37] = 0.002, *p* =.966), but there was a significant interaction between the time since the last meal × stimulus type (*F*[1.64, 60.59] = 6.28, *p* =.005, η^2^ = 0.082). Post hoc analysis with Bonferroni correction was performed to compare responses in the same stimulus category between groups. HC gave higher wanting ratings of high-calorie food compared to AN-R patients (*t*[37] = -2.13, *p* =.040, *d* = 2.13), whereas AN-R patients rated wanting nonfood objects more than HCs did (*t*[37] = 3.35, *p* =.002, *d* = 3.35). There were no differences in wanting ratings between groups for amodal foods (*t*[37] = -0.82, *p* =.419) and low-calorie foods (*t*[37] = 0.10, *p* =.918).

## Discussion

This study aims to build upon previous findings that individuals with AN-R exhibit changes in their approach and avoidance mechanisms when processing food-related stimuli. Specifically, it investigates whether patients engage with high-calorie stimuli at a higher construal level—interpreting and responding to these cues based on abstract dieting ideals rather than hedonic impulses. To test this hypothesis, we aimed to extend the results of Schroeder, Collantoni, et al. [7], who reported behavioral avoidance patterns toward food cues in patients with AN-R. Specifically, to better understand the influence of cue representation, we included amodal (packaged/named food) and low-calorie food in a VR SST. However, we unexpectedly found that participants reached the furthest to amodal food cues, whereas movements toward high-calorie and low-calorie concrete food cues were the shortest. Additionally, compared with the HCs, the AN-Rs showed lower wanting of high-calorie food items and greater wanting of nonfood (shoe) cues. Liking was not affected by group affiliation. However, high-calorie cues were liked the most, regardless of whether the cue presentation was amodal or concrete. Thus, one interpretation of the current data is that the presentation of food cues can affect approach behavior toward food cues, even if the subjective ratings do not differ within high-calorie cues as an overarching category.

In contrast to our expectations, we were unable to replicate the interaction between food and nonfood stimuli reported by Schroeder, Collantoni, et al. [7]. In their study, the authors reported increased avoidance in AN-R for food stimuli compared with nonfood objects. Here, we generally found a smaller displacement in patients with AN-R than in HCs without (high-calorie) food specificity. This contrasts previous studies that found reduced approach bias towards high-calorie, but not low-calorie food cues in AN [5], or a generally reduced approach towards food cues in AN compared to HC [6, 8, 45]. Although these studies collectively suggest that patients with AN-R may demonstrate reduced approach behavior specifically towards (high-calorie) food cues, the current study could not confirm this hypothesis.

One potential reason for this difference is the increased complexity of the current study. Here, compared to previous studies, we had more control conditions (amodal, low-calorie, neutral), which increased the task duration and changed the proportions between food and control stimuli. Our data suggest that participants effectively differentiated between modal and amodal stimuli during the SST, as shown in SST approach-avoidance behavior. Within the modal category, stimuli were predictive of food at more than the guessing rate (odds ratio 2:1). As a result, modal stimuli (i.e., unpackaged stimuli) were predictive of food cues. Particularly in patients with AN, this may have implemented a strategy of continuous avoidance, which could have led to smaller differences in approach behavior between food and nonfood stimuli. Moreover, the introduction of amodal stimuli in a design with randomized trials may have affected the processing of nonfood stimuli. Specifically, the simultaneous use of different sensory modalities in a single paradigm can alter spatial cognitive processing [46], although it is unknown how the sequential presentation of variations in a single sensory modality influences approach behavior and inhibitory control.

In light of construal level theory [14], amodal stimuli should be on a higher construal level than modal stimuli, as they display fewer concrete, sensory features [15]. Indeed, here, we find that both approach behavior and successful inhibition were greater for packaging with food names than for those with depicted food stimuli. With regard to our unexpected null finding, the contrast with amodal stimuli, which elicited an unexpectedly large effect, may have forced a distinction between low- (sensory) and high-construal-level processing (i.e., different levels of abstraction). This could have resulted in more similar processing of low-level construal stimuli, including our nonfood cues. Future studies are therefore needed to better discriminate food-specific effects. These comparisons should take place either in the absence of amodal cues or in block designs that prevent cross-modal activation of high- and low-construal-level concepts.

Taken on its own, the enhanced performance for amodal stimuli is remarkable since the cognitive liking and wanting ratings of the amodal stimuli were similar to those of the depictions of high-calorie food. One interpretation of this data is that, compared with sensory cues, abstraction of a task-irrelevant stimulus reduces interference with performance despite similar cognitive appraisals. Previous studies have linked the activation of higher construal-level thinking with reduced food intake [17–19], raising the question of whether such representations could be harnessed to support dietary restraint. However, our findings suggest that such mechanisms may not operate automatically. In our paradigm, where all stimuli were task-irrelevant, the effect of modal stimuli on reaching behavior was comparable between HCs and AN-R patients. This finding runs against a previous study that reported that stimulus modality differentially affected inhibitory control for food cues compared with nonfood cues in those with dietary restraint compared with those without [47]. By extrapolating this to AN-R, we may have expected different representations of amodal food compared with HCs. However, the current study lacks the power to detect a similarly small, food-specific modality effect in AN-R. Moreover, the current study did not have a nonfood amodal condition to determine whether this effect is specific to modality (e.g., pertaining to word processing) or to food cues. Further studies are therefore needed to extend the current findings, for example, by investigating whether abstraction is used by patients with AN-R to control behavior toward task-relevant stimuli.

Further considerations emerging from this study relate to some reflections from a therapeutic perspective. In clinical settings focused on psychotherapy and nutritional rehabilitation, exposure to food represents a critical component of treatment. The present findings suggest that graded exposure interventions might benefit from being calibrated along a continuum of stimulus abstraction, beginning with more abstract, amodal representations of food and progressively introducing more concrete and sensory-rich cues. This stepwise approach could reduce the initial emotional reactivity associated with high-calorie foods and facilitate engagement with avoided stimuli. Notably, research investigating the sensorimotor activation elicited by food stimuli in eating disorders remains limited. Future studies should further explore how different levels of sensory engagement influence both implicit behavioral responses and treatment outcomes in individuals with AN.

The current results need to be interpreted with the following considerations in mind. First, we did not reach the desired power for the contrasts of interest. Although we estimated the effect size conservatively on the basis of previous research [7], we may have failed to detect some true effects. Therefore, on the base of the current study our original hypotheses cannot be confidently rejected. Moreover, the low power may have led to an overestimation of the effect size of the found effects. Hence, the found effect sizes should be interpreted with caution. Additionally, although we did not find systematic exclusion effects in our patient group, there were differences in age and trait anxiety between in- and excluded participants in the control group (Table S2). This may have additionally introduced a bias in the results and further limits the generalizability. A replication of the current work with an increased sample size, and improved methodology against drop-out (i.e., automated feedback) is therefore necessary to verify the study’s outcome. Second, as previously mentioned, the present protocol lacks a nonfood amodal condition. As increased abstraction of food cues should be associated with loss of salient, sensory cues according to the modal and amodal theory of mental representation [15], any food-specific effects should be diminished for amodal stimuli. As we currently did not find differences between food and nonfood stimuli for the depicted stimuli, we would therefore not expect any differences between amodal cues. Nevertheless, the absence of the condition in the current study means that we cannot say if this is particular to food cues. Third, we did not systematically collect (psychological) comorbidities in our patient or control sample. Especially with regard to depressive symptoms this impedes the estimation to what degree general motivational deficits may have influenced our results [48]. However, these factors are difficult to disentangle, as depression rates are generally higher in AN populations [49]. Fourth, the current sample was exclusively female and recruited over a select number of clinics, limiting sample diversity. This is further exacerbated by missing social economic status and ethnicity data. Additionally, at the time of the study, a structural consideration of lived experiences’ perspectives was not implemented in the study due to lack of resources, but according infrastructures are currently being promoted [50]. Future research is therefore required to determine generalizability of our findings. Finally, the current study did not standardize the participants’ level of satiation. Owing to regular meals in the stationary setting, patients with AN-R had their last meal closer to participating in the experiment and experienced less hunger. Although we controlled for this in our analyses, it is difficult to attribute the current findings to a particular homeostatic state. Since hunger can influence attentional processes [51] and modality-dependent inhibitory control [44], future studies with HCs may manipulate satiation to uncover the effect of homeostatic state on the VR SST.

## Conclusion

The current study investigated how mental representations of food cues influence approach and inhibitory behavior in patients with AN-R and HCs. We found that approach and stop behavior were enhanced for amodal (packaged/named) food stimuli in comparison with unpackaged, high-fidelity depictions of food and nonfood items in both AN-R patients and HCs. This is in line with a higher construal representation, in which task-irrelevant features are not represented and therefore do not interfere with task performance. In general, higher construal level thinking is associated with lower food intake [17–19]. Future studies may therefore focus on the use of methods that may lead to higher construal levels, such as (psychological) distancing [14], and whether these methods (temporarily) aid food restraint in AN-R patients and healthy controls.

### Secondary variables

We used ANCOVA to determine the effects of Group and Stimulus type on the false alarm rate in stop trials. Our analysis revealed a main effect of Stimulus type (*F*[1,111] = 61.30, *p* <.001, η^2^ = 0.25) in the absence of the other main and interaction effect (*F*s < 2.25, *p*s > 0.086; Figure S1). Follow-up paired sample *t*-tests indicated that there were differences between amodal and nonfood (*t*[39] = -12.33, *p* <.001, *d* = 1.95) and amodal and high-calorie stimuli (*t*[39] = -9.00, *p* <.001, *d* = 1.42). For these comparisons, amodal stimuli had a lower false alarm rate than high-calorie or nonfood stimuli did. Neither the comparison between high- and low-calorie stimuli (*t*[39] = 0.35, *p* =.729) nor the comparison between high-calorie and nonfood stimuli (*t*[39] = -0.78, *p* =.443) were significant.

Similarly, we used ANCOVA to determine the effects of Group and Stimulus type on the reaction times in successful go trials. This indicated a main effect of Group (*F*[1,37] = 7.70, *p* =.009, η^2^ = 0.16) and Stimulus type (*F*[1,111] = 87.13, *p* <.001, η^2^ = 0.18) but no interaction (*F*[1,37] = 2.54, *p* =.060). Compared with the healthy controls, the AN-R group generally had longer reaction times. Analogous to the false alarm rates, follow-up paired sample *t*-tests indicated that there were differences between amodal and nonfood (*t*[39] = 9.81, *p* <.001, *d* = 1.55) and amodal and high-calorie stimuli (*t*[39] = 10.20, *p* <.001, *d* = 1.61). For these comparisons, amodal stimuli had a shorter reaction time than did high-calorie and nonfood stimuli. Neither the comparison between high- and low-calorie stimuli (*t*[39] = 0.58, *p* =.568) nor the comparison between high-calorie and nonfood stimuli (*t*[39] = -0.83, *p* =.412) were significant.

To test for differences in movement initiation times, we used a 2 × 2 × 4 ANCOVA. This analysis indicated a main effect of Group (*F*[1,37] = 12.20, *p* =.001, η^2^ = 0.24) in the absence of other main and interaction effects (*F*s < 2.08, *p*s > 0.106). The effect of Group demonstrated a later onset of movement in AN-R patients than in HCs.

## Supplementary Information

Below is the link to the electronic supplementary material.

Supplementary Material 1

## Data Availability

Data and analyses are available at https://osf.io/acxkg/.

## Abbreviations

AN-R: Anorexia nervosa, restrictive subtype
BMI: Body mass index
EDE-Q: Eating Disorder Examination Questionnaire
HC: Healthy control
SST: Stop signal task
STAI: State-Trait Anxiety Inventory
VAS: Visual Analog Scale
VR: Virtual Reality
