# Supplementary material for: Psychological distance affects real movements in virtual reality: distance to food in anorexia nervosa
Source: J Eat Disord. 2025 Aug 6;13:169. doi: 10.1186/s40337-025-01357-0 (PMC12329868; doi:10.1186/s40337-025-01357-0)
Supplement: Supplementary file 1 — Supplementary Material 1 [file 40337_2025_1357_MOESM1_ESM.docx]

**Psychological distance affects real movements in virtual reality: Distance to food in anorexia nervosa**

Mechteld M. van den Hoek Ostende^1^, Giulia Brizzi^2,3^, Valentina Meregalli^,5^, Philipp A. Schroeder^1,6^ & Enrico Collantoni^4^

^1^   Department of Psychology, Clinical Psychology & Psychotherapy, University of Tübingen, Tübingen, Germany

^2^  Department of Psychology, Università Cattolica del Sacro Cuore, Milan, Italy

^3^ Humane Technology Laboratory, Università Cattolica del Sacro Cuore, Milan, Italy

^4^ Department of Neuroscience, University of Padova, Padova, Italy

^5^ Department of General Psychology, University of Padova, Padova, Italy

^6^ German Center for Mental Health (DZPG), partner site Tübingen

**Supplementary Materials**

**Comparison drop-outs: AN-R**

*Table S1.* Comparison in-and excluded participants, AN-R group

| **Variable** | **Group** |  | **Group comparison** |
| --- | --- | --- | --- |
|  | **Included (*n*= 21)** | **Excluded (*n*= 7)** |  |
| Age | 17.1 (2.86) | 18.4 (3.55) | *t*(8.8) = -0.900, *p* = .39 |
| BMI | 16.4 (1.22) | 15.3 (1.33) | *t*(9.6) = 2.06, *p* = .067 |
| Hunger | 1.35 (0.93) | 1.29 (0.49) | *t*(20.6) = 0.231, *p* = .82 |
| EDE-Q total | 3.88 (1.33) | 3.18 (1.64) | *t*(8.8) = 1.02, *p* = .336 |
| STAI trait | 63.5 (8.47) | 57.3 (16.1) | *t*(7.1) = 0.983, *p* = .358 |
| UPPS total | 8.40 (1.77) | 8.3 (1.40) | *t*(13.0) = 0.174, *p* = .864 |

## AN-R = patients diagnosed with anorexia nervosa, restrictive type; BMI = body mass index; EDE-Q = Eating Disorder Examination Questionnaire; STAI = State-Trait Anxiety Inventory; UPPS = UPPS Impulsive Behavior Scale

**Comparison drop-outs: HC**

*Table S2.* Comparison in-and excluded participants, HC group

| **Variable** | **Group** |  | **Group comparison** |
| --- | --- | --- | --- |
|  | **Included (*n*= 19)** | **Excluded (*n*= 8)** |  |
| Age | 18.1 (4.37) | 22.6 (4.47) | *t*(13.0) = -2.42, *p* = .031 |
| BMI | 21.2 (1.78) | 20.5 (0.94) | *t*(23.4) = 1.36, *p* = .188 |
| Hunger | 3.21 (1.90) | 3.14 (2.41) | *t*(8.9) = 0.067, *p* = .948 |
| EDE-Q total | 0.84 (0.89) | 0.40 (0.33) | *t*(24.4) = 1.87, *p* = .074 |
| STAI trait | 49.3 (8.54) | 39.5 (9.72) | *t*(11.8) = 2.48, *p* = .029 |
| UPPS total | 9.51 (2.08) | 8.1 (1.50) | *t*(18.2) = 1.93, *p* = .069 |

## BMI = body mass index; EDE-Q = Eating Disorder Examination Questionnaire; HC = healthy controls; STAI = State-Trait Anxiety Inventory; UPPS = UPPS Impulsive Behavior Scale

**Supplementary materials**

Low-calorie foods

https://sketchfab.com/3d-models/apple-643eb66651864bb78871e5c1066b4ef6

https://sketchfab.com/3d-models/low-poly-lettuce-a10d2ab6070f4d95adad6bf453179141

https://sketchfab.com/3d-models/cara-cara-orange-ad325912dc614f1596d2dad2d64524eb

https://sketchfab.com/3d-models/papryka-lp-94032d9c7b1746079828b54b5d02c533

https://sketchfab.com/3d-models/rice-bowl-24464015c15b4662a32be7625ae3f75b

Amodal packaging:

https://sketchfab.com/3d-models/happy-meal-fast-food-package-9639060eb0ae4c82be37443bbd75435c

**Supplementary Figure 1**


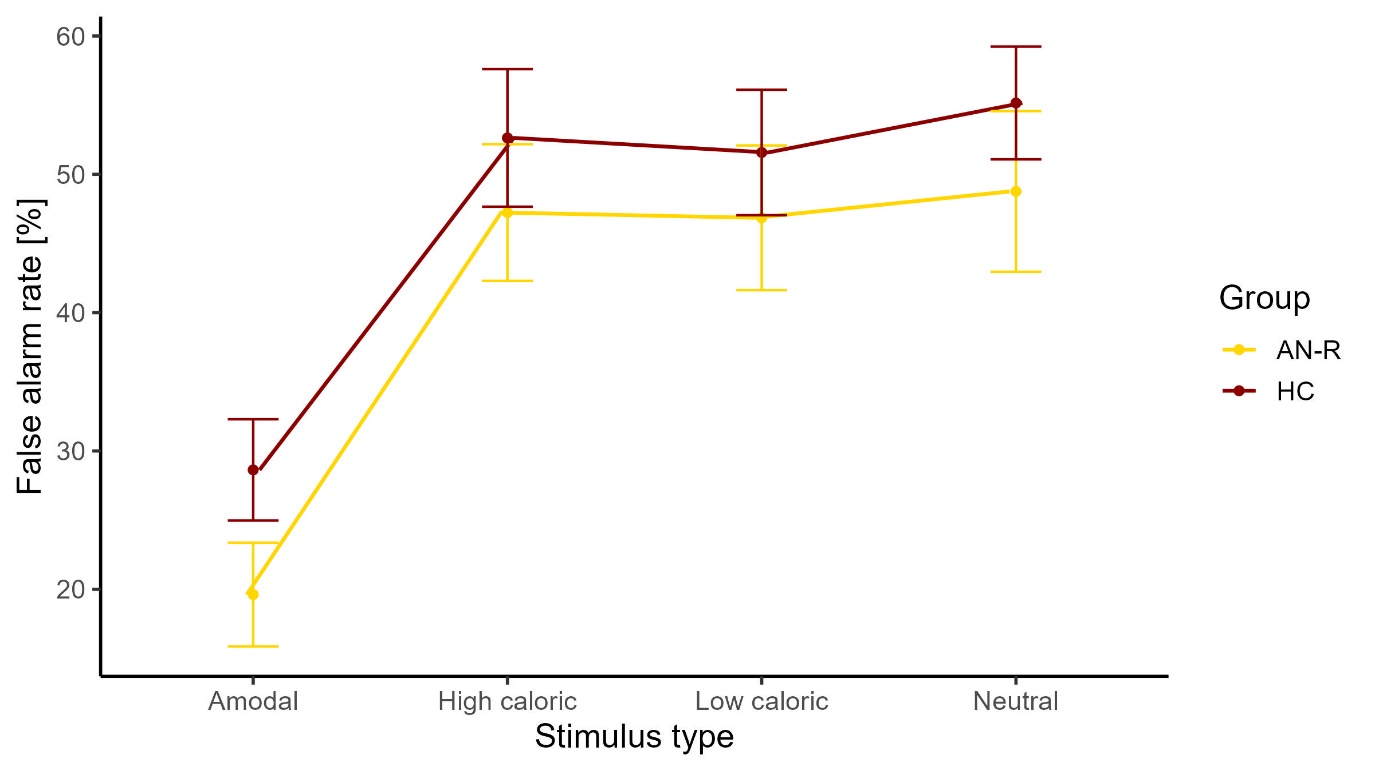


*Figure S1*: False alarm rates for stop trials. False alarm rates are lowest for amodal stimuli for both anorexia nervosa, restrictive type (AN-R) and healthy controls (HC). Error bars indicate standard errors.
